# Neuropathic Pain Was Associated with Central Sensitivity Syndrome in Patients with Preoperative Lumbar Spinal Stenosis Using the painDETECT and Central Sensitization Inventory Questionnaires: A Cross-Sectional Study

**DOI:** 10.1155/2023/9963627

**Published:** 2023-05-10

**Authors:** Rintaro Iwasaki, Takahiro Miki, Mizuki Miyazaki, Chifumi Kanetaka, Tetsuryu Mitsuyama, Kaiji Ota

**Affiliations:** ^1^Department of Rehabilitation, Shisyokai Hakusan Clinic, Tokyo, Japan; ^2^Department of Rehabilitation, Sapporo Maruyama Orthopedic Hospital, Sapporo, Hokkaido, Japan; ^3^Faculty of Health Sciences, Hokkaido University, Sapporo, Hokkaido, Japan; ^4^Department of Rehabilitation, Shinagawa Shisyokai Hospital, Tokyo, Japan; ^5^Department of Neurosurgery, Shinagawa Shisyokai Hospital, Tokyo, Japan; ^6^Department of Orthopedic Surgery, Shinagawa Shisyokai Hospital, Tokyo, Japan

## Abstract

**Background:**

Lumbar spinal stenosis (LSS) patients have been reported to have neuropathic pain and central sensitivity syndrome (CSS). These associations have been reported in other diseases but are unknown in preoperative LSS patients. We aimed to investigate the association between neuropathic pain and CSS in preoperative LSS patients using the painDETECT and the Central Sensitization Inventory (CSI) questionnaires.

**Methods:**

This cross-sectional study was conducted from November 2021 to March 2022. The data were collected regarding demographics and pain, including neuropathic pain, numbness, LSS severity, physical function, quality of life, and CSS. Patients were divided into two groups, patients with acute and chronic pain, and further classified into three categories based on the clinical phenotype of patients in each group. Independent variables included age, gender, type of LSS (bilateral or unilateral symptoms), Numerical Rating Scale of leg pain, CSI, and the Zurich Claudication Questionnaire (ZCQ) for symptom severity and physical function. The dependent variable was painDETECT. Multiple regression analysis using the forced entry method examined the association between painDETECT and CSI.

**Results:**

Of the 119 patients with preoperative LSS, 106 were included. The mean age of the participants was 69.9 years, and 45.3% were female. Neuropathic pain was present in 19.8%, and CSS was present in 10.4%. The CSI (*β* = 0.468, *p* < 0.001) and ZCQ for symptom severity (*β* = 0.304, *p* < 0.01) were significantly associated with the painDETECT, explaining 47.8% of the variance in the painDETECT score.

**Conclusions:**

There is an association between neuropathic pain and CSS in patients with preoperative LSS using the painDETECT and CSI questionnaires.

## 1. Introduction

Lumbar spinal stenosis (LSS) is a degenerative disease impacting daily living and quality of life, with neuropathic pain being the primary complaint [1, 2]. A previous study using the painDETECT, which can screen for neuropathic pain, reported that 17.6% of LSS patients were classified as having neuropathic pain [3]. On the other hand, Miki et al. mentioned that 13% of patients with preoperative LSS and lumbar disc herniation had central sensitivity syndrome (CSS) [4]. Central sensitization (CS) is an essential element in understanding CSS. CS exhibits abnormal nociceptive systems with the enhancement of nociceptive pathways resulting from significant somatosensory nervous system plasticity [5]. CS and CSS are symptoms without a specific organic cause [6]. Neuropathic pain and CSS involve abnormalities in the somatosensory system and may have similar clinical manifestations [7, 8]. Furthermore, Akeda et al. stated that CSS might be associated with neuropathic pain symptoms caused by central or peripheral nervous system damage due to spinal disease [9]. For that reason, it is reported that CSS features have often been interchanged with neuropathic pain terms, leading to confusion [10]. In this way, the heterogeneity of the clinical symptoms and pathophysiology of LSS contribute to the difficulty in understanding the source of pain [2]. Additionally, associations between neuropathic pain, CSS, and chronic pain have been reported, highlighting the importance of investigating these relationships for pain management and treatment in LSS patients [11–14]. Despite the association between neuropathic pain and CSS, no studies have investigated the potential association between the two in patients with preoperative LSS. The investigation of these relationships will lead to an understanding of the mechanisms underlying pain. In addition, neuropathic pain and CSS have different treatment options, but there is potential to contribute to developing more effective treatments [15, 16]. Thus, investigating these potential associations would be beneficial for pain management and treatment, and this study is the first attempt to investigate preoperative LSS patients in this area.

In this study, we aimed to examine preoperative LSS patients for potential associations between neuropathic pain and CSS, hypothesizing that neuropathic pain and CSS would not be related. Rejecting this null hypothesis would imply a latent relationship between neuropathic pain and CSS, providing valuable insights for managing and treating pain in LSS patients.

## 2. Methods

### 2.1. Study Design and Setting

This cross-sectional study was conducted between November 2021 and March 2022 at Shinagawa Shishokai Hospital. The study was approved by the Shinagawa Shishokai Hospital Research Ethics Committee (reference no. 202101) and conducted in compliance with the Declaration of Helsinki. All participants provided written informed consent to participate following a comprehensive discussion about study procedures and objectives. The study methods were compliant with the STROBE checklist [17].

### 2.2. Participants

We included patients (1) who were hospitalized for surgery after being diagnosed with LSS, (2) who agreed to participate in the study, and (3) who were able to read the Japanese language well enough to complete the questionnaires independently. We excluded the following patients: (1) those with histories of musculoskeletal disorders and (2) those who did not complete the questionnaires.

### 2.3. Variables

We collected data on age, gender, height, weight, body mass index (BMI), duration of LSS symptoms, and details of conservative treatments before surgery from medical records. Patients reported the duration of LSS symptoms in months or years. If the period was in years, it was converted to months. Chronic pain is pain that lasts or recurs for over three months [18]. This study classified LSS symptoms lasting more than three months as chronic pain and those lasting less than three months as acute pain. The type of LSS was classified into bilateral or unilateral symptoms. LSS symptoms and radiological severity were assessed. In addition, we collected data on pain intensity, pain quality, CSS, LSS symptom severity, physical function, and quality of life by patient-reported outcome measures (PROMs). Before surgery, patients completed all PROMs. The outcome measures are described in more detail in the following.

A taxonomy based on clinical phenotype was used to assess LSS symptoms. Based on the clinical phenotype of the patients, we classified them into three categories: Type A, neurogenic claudication pain; Type B, neurogenic claudication sensory/balance; and Type C, radicular unilateral leg pain, based on the report of Comer et al. [19].

The Schizas classification was used to assess the radiological severity of LSS [20]. The participants were graded A (A1–A4), B, C, and D according to the classification by Schizas, which grades the cerebrospinal fluid ratio to nerve roots and spinal canal on a T2-weighted MRI scan of the spine. The highest grade level was considered for the analysis of multiple spinal stenoses. The planned surgical site determined the number and location of spinal stenosis.

The Numerical Rating Scale (NRS) was used to rate the average low back, leg, and numbness intensity on the assessment day. A score of 0 indicated no pain, and 10 indicated the worst pain imaginable.

The painDETECT was used to screen for neuropathic pain [21]. This questionnaire consists of nine items (seven pain-symptom items, one pain course, and one pain irradiation) completed on different scales. The cutoff values for pain categorization were nociceptive pain, 0–12; unclear pain (mixed nociceptive and neuropathic pain), 13–18; and neuropathic pain, 19–38. The questionnaire reliability was previously confirmed (Cronbach's alpha = 0.83). The painDETECT sensitivity is 84% and specificity is 84%. The Japanese version of the painDETECT is considered reliable and valid with a Cronbach's alpha of 0.80 and an intraclass correlation coefficient (ICC) of 0.943 [22].

The Central Sensitization Inventory (CSI) was used to evaluate CSS. The CSI is a self-report questionnaire that does not determine causality but can assess CS-related symptoms. The CSI comprises 25 items with scores ranging from 0–4 [23]. The higher the total score, the more significant the CS deficits; a total score of 40 is considered the cutoff point. A cutoff score of 40 out of 100 on the CSI has demonstrated excellent sensitivity (81%) for correctly identifying patients with CSS and acceptable specificity (75%) for correctly identifying nonpatient comparison subjects [24]. In this study, a score of 39 or lower was considered a low CSI, and a score of 40 or higher was considered high [25]. The Japanese version of the CSI is reliable and valid, with a Cronbach's alpha of 0.89 and an ICC of 0.85.

The Zurich Claudication Questionnaire (ZCQ) was used to evaluate the LSS comprehensively [26]. The 12-item ZCQ was developed to evaluate LSS comprehensively. The symptom severity consists of 7 items with scores ranging from 1–5, and physical function consists of 5 items with scores ranging from 1–4. ZCQ scores range from 12–55, with higher scores indicating more dysfunction. The Japanese version of the ZCQ is reliable and valid, with Cronbach's alphas of 0.78 (symptom severity) and 0.84 (physical function) and ICCs of 0.81 (symptom severity) and 0.89 (physical function) [27].

The EQ-5D-5L was used to assess quality of life (QoL) [28]. The Japanese version of the EQ-5D-5L consists of a 1 five-item, five-point categorical scale commonly used to measure the QoL. The total score ranges from −0.025–1. A higher score on the EQ-5D-5L indicates good QoL. The utility values are calculated from response integers using the formula reported by Ikeda et al.

### 2.4. Outcomes

The primary outcome was painDETECT and CSI. Multiple regression analysis was performed to evaluate these associations with painDETECT as the dependent variable and CSI and other items as independent variables. Details are described in the Statistical Variables section.

### 2.5. Bias

Several strategies were implemented to minimize bias. All PROMs utilized standardized questionnaires with established reliability and validity in Japanese. Patients who required assistance comprehending the questionnaires or had incomplete data were excluded to guarantee a comprehensive data analysis. Moreover, the assessment date was designated as the day before surgery. Patients were informed that the evaluator and therapist might not be the same individual, ensuring that their questionnaire responses would not adversely affect their treatment.

### 2.6. Study Size

The sample size was calculated using *G*^*∗*^Power (version 3.9.6.1) [29, 30]. G^∗^Power uses the a priori power analysis method to calculate the necessary sample size based on the selected statistical analysis, desired power, and other input parameters. We entered the effect size, *α* error, power, and the number of predictors into the *F* tests to calculate the sample size. The statistical test used was a linear multiple regression with a fixed model, focusing on R^2^ deviation from zero. The power analysis type involved calculating the required sample size based on *α*, power, and effect size.” Specifically, we set the effect size *f*2 to 0.15, *α*err prob to 0.05, power to 0.8, and the number of predictors to 7, as Cohen [19] recommended for a medium effect size in psychological research. The resulting sample size was 103 participants.

### 2.7. Quantitative Variables

Participants' characteristics were summarized with descriptive statistics. Quantitative variables were presented for mean, standard deviation, minimum, maximum, median, interquartile range, and 95% confidence interval. Counts and percentages summarized categorical variables.

### 2.8. Statistical Variables

For all variables, the Shapiro–Wilk test was used to assess the normality of the data distribution. For comparing continuous variables in the two acute and chronic pain groups, independent *t*-tests were used for variables meeting the normality assumption (*p* > 0.05). Otherwise, the Mann–Whitney *U* test was used. Categorical variables were examined with a chi-square test (expected frequency >5) or Fisher's exact probability test. Independent *t*-tests were used for the following items: height, painDETECT, ZCQ, ZCQ for symptom severity, and ZCQ for physical function. Independent Mann–Whitney *U* tests were used for the following items: age, weight, BMI, duration of LSS symptoms, number of spinal stenosis, NRS for LBP, leg pain, numbness, CSI, and EQ-5D-5L. Independent chi-square tests were used for the following items: gender. Independent Fisher's exact test was used for the following items: classification of LSS by the site of symptom appearance, grade of spinal stenosis according to Schizas, and conservative treatment. Two groups, acute and chronic pain, were classified into three types, each using a clinical phenotype. Each factor's correlation was evaluated using Spearman's rank correlation coefficient (*r*). In addition, multiple regression analysis using the forced entry method was performed using painDETECT as the dependent variable and age, gender, and grade of classification of LSS by the site of symptom appearance, CSI, ZCQ for symptom severity, and ZCQ for physical function as independent variables to determine which factors affected the painDETECT score. Regarding multicollinearity, we confirmed that the analysis of variance (VIF) for all independent variables was <5. All statistical analyses were performed using EZR (Saitama Medical Center, Jichi Medical University, Saitama, Japan) and GUI of R (the R Foundation for Statistical Computing, Vienna, Austria).

## 3. Results

### 3.1. Participants' Characteristics

We screened 119 participants with LSS; 13 (8%) were excluded due to previous surgery (*n* = 9), inability to understand (*n* = 1), difficulty understanding Japanese (*n* = 1), and incomplete data (*n* = 2). Finally, 106 participants satisfied all criteria and agreed to participate. Characteristics of the participants and clinical features of the total sample are shown in Table 1. A comparison of the demographic and clinical characteristics of acute and chronic pain is shown in Table 2. Significant differences were observed in gender, duration of LSS symptoms, classification of LSS by site of symptom appearance, number of spinal stenosis, and NRS for LBP.

### 3.2. Bivariate Correlation Analysis

Table 3 shows the correlation matrix for all participants. The painDETECT was positively associated with the NRS for low back pain, leg pain, and numbness (*r* = 0.341, *p* < 0.001,*r* = 0.428, *p* < 0.001, *r* = 0.429, *p* < 0.001), CSI (*r* = 0.550, *p* < 0.001), ZCQ for symptom severity (*r* = 0.523, *p* < 0.001), and ZCQ for physical function (*r* = 0.308, *p* < 0.001) and negatively associated with the EQ-5D-5L (*r* = −0.426, *p* < 0.001). The CSI was positively associated with the NRS for low back pain, leg pain, and numbness (*r* = 0.444, *p*0.001, *r* = 0.327, *p* < 0.001, *r* = 0.293, *p* < 0.001), ZCQ for symptom severity (*r* = 0.307, *p* < 0.001), and ZCQ for physical function (*r* = 0.305, *p* < 0.001) and negatively associated with the EQ-5D-5L (*r* = −0.461, *p* < 0.001).

### 3.3. Multiple Regression Analysis

The multiple regression analysis was performed using a forced entry method. The CSI (*β* = 0.468, *p* < 0.001) and ZCQ for symptom severity (*β* = 0.304, *p* < 0.01) were significantly associated with the painDETECT, explaining 47.8% of the variance (*r*^2^ adjusted: 0.478, Table 4).

## 4. Discussion

This was the first study to investigate the potential association between neuropathic pain and CSS in preoperative patients with LSS using painDETECT and CSI. The results showed a significant association between the painDETECT and CSI by multiple regression analysis. Furthermore, we also found an excellent convergent association between painDETECT and CSI, as 47.8% of the variance was explained. These findings confirm the association between neuropathic pain and CSS in preoperative patients with LSS. The painDETECT and CSI were nonsignificant between the two groups for acute and chronic pain. No significant association was found between painDETECT and CSI in classifying LSS according to clinical phenotype.

Similar to previous studies, leg symptoms in LSS included a neuropathic pain component in this study [2]. As leg symptoms in LSS result from the narrowing of the spinal canal, neuropathic pain is unlikely to influence symptom duration. Therefore, we consider that the difference between the two groups due to acute and chronic pain was nonsignificant. CSS is associated with CS, which is more common in patients with chronic pain [31]. The mechanisms of CSS require further understanding. CSS has been observed even with nonpainful stimulation, and it has been reported that pain augmentation is unlikely to be a significant causal factor in CSS [6]. Since pain is unlikely to trigger CSS, we consider the difference between the two groups concerning acute and chronic pain nonsignificant. Additionally, the nonsignificant difference between the two groups may be attributed to the screening tools not containing questions related to symptom duration. We also speculate that the difference between the two groups is nonsignificant because these screening tools do not have questions related to the duration of symptoms. The classification of LSS by clinical phenotype is a recently developed classification method. For this reason, no investigation of each factor using this classification method has been conducted to date. In a previous study, patients with LSS were divided into two groups, one with predominant radicular pain and the other with predominant neurogenic intermittent claudication (NIC). The group with predominant radicular pain had a higher LANSS score in identifying neuropathic pain [2]. However, the present study found an association between clinical phenotype and painDETECT. Although the creation mechanism of NIC development has not been elucidated, it is currently thought to be due to decreased blood flow to the nerve root and venous stasis [1, 32]. Furthermore, leg pain in LSS is caused by nerve root compression, classified as neuropathic pain. Therefore, we consider that the clinical phenotypes classified into three types were not significantly different in painDETECT. In that study, patients with radicular pain that did not improve upon flexion were classified into the radicular pain group. In this study, however, the classification is based on the clinical phenotype of the patients. In addition, painDETECT uses a five-point Likert scale, while LANSS uses a two-point Likert scale. Differences in the criteria for classifying patients with LSS and the screening tool used led to differences in the results from previous studies. CSI also showed no significant association with clinical phenotype. Tanaka et al. reported that CSI is a clinical utility prediction tool for CSS regardless of the type of diagnosis [33]. Therefore, we speculate that although there is a certain number of CSS in the disease LSS, we did not find significant differences by symptom because there is no organic cause.

Furthermore, in a previous study in which patients with LSS were classified by painDETECT, 17.6% had neuropathic pain [3], and 17.9% of the study subjects had neuropathic pain. Furthermore, in a previous study of patients with preoperative LSS and lumbar disc herniation, 13.1% had high CSI [4], and 10.4% of the subjects had high CSI. Both neuropathic pain and CSI showed similar rates as in previous studies. The reason for this is considered to be a similar patient background. Similar to other conditions, this study confirmed a potential link between painDETECT and CSI [11, 12]. The significant association between painDETECT and CSI in patients with preoperative LSS is a new finding. Two studies examined the association between the painDETECT and CSI. The first study, involving participants recruited from the Spanish Fibromyalgia Association, consisted of women with fibromyalgia who had scores of 19.9 ± 7.1 and 70.7 ± 11.6 for painDETECT and CSI, respectively [12]. Another study was of outpatients on long-term follow-up for rheumatoid arthritis with scores of 7.48 ± 5.21 and 18.3 ± 11.8 for painDETECT and CSI, respectively [11]. The previous study, as well as the present study, showed an association between painDETECT and CSI. However, there are differences in painDETECT and CSI scores for each disease. The mechanism of the disease may influence the reason for this. LSS affects the cauda equina, epidural pressure, and nerve roots by reducing the spinal canal and foraminal space [34], and nerve root compression has been reported to cause neuropathic pain [35]. Therefore, neuropathic pain was considered to be observed in the subjects of this study. CSS has been defined as symptoms with no specific organic cause but is associated with CS [36]. It is currently unknown whether pain augmentation affects CSS [6]. The appearance of primary and secondary hyperalgesia characterizes neuropathic pain. An increased response to stimuli is primary hyperalgesia. Secondary hyperalgesia is thought to be caused by sensitization of the central nervous system [37]. It has also been reported that peripheral nerve damage can lead to CS [38]. Therefore, CSS was considered to be observed in the subjects of this study. In addition, two studies used the painDETECT to investigate signs of CS. One study included preoperative patients with hip osteoarthritis. Functional magnetic resonance imaging was used to evaluate the sharpness of point stimuli and changes in brain activity that occurred in response to stimulation [39]. Patients with high painDETECT scores (above the sample median) were likelier to show signs of CS. Another study included patients with knee osteoarthritis and higher modified painDETECT scores. Their results revealed a lower pressure threshold for pain when assessing CS [29]. Therefore, painDETECT scores appear to be associated with signs of CS. Also reported was a possible association between neuropathic pain and CSS in patients with rheumatoid arthritis and patients undergoing elective spinal surgery [9, 11, 30]. Furthermore, it has been reported that medication restored or enhanced the descending pain suppression system in patients with chronic nerve root pain and diabetic polyneuropathy [40, 41]. Descending pain inhibitory systems have been involved in CS [42, 43], and CS is observed in neuropathic pain [44]. These reports may further emphasize the overlapping mechanisms and associations between neuropathic pain and CSS. Unfortunately, the mechanisms linking neuropathic pain and CSS are yet to be elucidated.

However, an essential reason for the significant association between the painDETECT and the CSI might be the overlap between each other's questions. The questions “Does your pain radiate to other regions of your body?” in painDETECT and “I feel pain all over my body” in CSI are considered overlapping. In addition, painDETECT provides seven questions on pain symptoms. CSI has provided a question on “My muscles feel stiff and achy,” “My legs feel uncomfortable and restless when I am trying to go to sleep at night,” and “I have pain in my pelvic area.” Patients may have responded to these question scales as identical. Therefore, caution is needed in interpreting the relevance of these questionnaires. The exact mechanism by which compression of the lumbar spinal canal and neural foramen manifests as symptoms is not yet understood [1]. Therefore, the key to managing LSS, in which pain is the main symptom, is a comprehensive pain assessment.

One of the limitations of this study is that the subjects were only patients with preoperative LSS, which does not allow for application to primary care or postoperative patients. Because we only examined preoperative patients with LSS from a single institution, our results may need to be more generalizable to other patient groups or settings. Furthermore, LSS has been reported to present with various symptoms, including NIC and LP, with different symptom mechanisms. However, the present study was not conducted on a symptom-by-symptom basis. Future surveys by symptom type would yield new results. Second, neuropathic pain and CSS were assessed using painDETECT and CSI. Diagnosing neuropathic pain requires detailed assessment, including neurologic examination [45]. CSS is also a symptom associated with CS, and Quantitative Sensory Testing (QST) is used to assess CS [46, 47]. Therefore, neuropathic pain and CSS are inadequately assessed. However, the present study aimed to investigate potential associations of the screening assessment using a questionnaire. Therefore, the questionnaires were standardized. Third, neuropathic pain and CSS have been associated with psychosocial factors [48], none of which were assessed in our study. Many CSI items have been reported to be common elements of anxiety and depression [33]. Furthermore, patients with higher CSI scores have reported improved depressive symptoms in chronic spinal pain disorders with a program that included education, counseling, and physical training [49]. The association between neuropathic pain and CSS might have been better investigated if psychosocial factors were included. Finally, our data were cross-sectional and therefore captured one moment in time. Therefore, causal relationships cannot be confirmed.

## 5. Clinical Implication

First-line treatment for neuropathic pain is pharmacologic therapy. When ineffectiveness is not expected, invasive approaches are considered [15]. A multidisciplinary approach that includes physical, cognitive, and pharmacotherapy is recommended as the first-line treatment for CSS [16]. The present study suggests an association between neuropathic pain and CSS in patients with preoperative LSS evaluated. Therefore, symptoms of LSS that have been judged as neuropathic pain may have aspects of a CSS. Interventions for CSS and neuropathic pain may improve symptoms of LSS.

## 6. Conclusion

This study investigated the potential association between neuropathic pain and CSS in patients with preoperative LSS using painDETECT and CSI. These results suggest neuropathic pain was associated with CSS in patients with LSS preoperatively assessed by painDETECT and CSI.

## Figures and Tables

**Table 1:** Clinical data of participants.

	Mean (SD)	Range	Median (IQR)	95% CI
*Baseline variable*				
Age (years)	69.9 (13.8)	27–97	72 (63.3–79.8)	67.2–72.6
Gender: no. (%)				
Male/female	58 (54.7)/48 (45.3)			
Height (m)	1.60 (0.10)	1.34–1.84	1.61 (1.51–1.67)	1.58–1/62
Weight (kg)	64.2 (15.9)	37 - 128.4	62.1 (54.9–72.5)	61.1–67.3
BMI	25.0 (4.7)	15.8–44.2	24.25 (22.2–26.8)	24.1–25.9
*Clinical characteristics*				
Duration of LSS symptoms (month)	29.7 (46.9)	1–240	12 (3–36)	20.7–38.7
Classification of pain by course: no. (%)				
Acute pain/chronic pain	29 (27.4)/77 (72.6)			
Classification of LSS by site of symptom appearance: no. (%)				
Bilateral symptom	69 (65.1)			
Unilateral symptom	37 (34.9)			
Clinical phenotype of LSS: no. (%)				
Type A	29 (27.4)			
Type B	36 (34)			
Type C	31 (38.7)			
Number of spinal stenosis	2.1 (0.9)	1–5	2 (1–3)	1.9–2.3
Location of spinal stenosis: no. (%)				
L1/2	4 (3.8)			
L2/3	22 (20.8)			
L3/4	59 (55.7)			
L4/5	86 (81.1)			
L5/S	53 (50.0)			
Grade of spinal stenosis according to Schizas: no. (%)				
A2/A3/A4	5 (4.7)/2 (1.9)/7 (6.6)			
B	8 (7.5)			
C	45 (42.5)			
D	39 (36.8)			
Conservative treatment: no. (%)				
No therapy	5 (4.7)			
Medicine	80 (75.4)			
Infusion·injection	10 (9.4)			
Block	39 (36.8)			
Rehabilitation	29 (27.4)			
Massage	34 (32.1)			
Acupuncture	17 (16.0)			
NRS for LBP (0–10 range)	5.3 (2.8)	0–10	6 (3–7)	4.8–5.8
NRS for leg pain (0–10 range)	5.1 (2.9)	0–10	5 (3–8)	4.5–5.7
NRS for leg numbness (0–10 range)	4.6 (3.2)	0–10	4.5 (2–7.8)	4.0–5.2
painDETECT (−1–38 range)	13.5 (6.1)	0–33	12.5 (9.3–18)	12.3–14.7
Nociceptive pain: no. (%)	53 (50.0)			
Type of unclear: no. (%)	32 (30.2)			
Neuropathic pain: no. (%)	21 (19.8)			
CSI (0–100 range)	24.4 (13.3)	2–63	23 (16–32)	21.8–27.0
High CSI: no. (%)	11 (10.4)			
Low CSI: no. (%)	95 (89.6)			
ZCQ (12−55 range)	33.6 (6.0)	13–51	34 (29–38)	32.4–34.8
Symptom severity (7–35 range)	21.5 (4.5)	8–32	24 (19–24)	20.6–22.4
Physical function (5–20 range)	12.2 (3.2)	5–19	12 (10–14)	11.6–12.8
EQ-5D-5L (−0.025–1 range)	0.513 (0.219)	0.035–0.873	0.573 (0.344–0.670)	0.471–0.555

SD: standard deviation; CI: confidence interval; BMI: body mass index; NRS: Numerical Rating Scale; CSI: Central Sensitization Inventory; ZCQ: Zurich Claudication Questionnaire; EQ-5D-5L: EuroQol-5 dimensions-5 levels. The range represents the minimum and maximum values. IQR represents the interquartile range.

**Table 2:** Clinical data of participants classified by duration of lumbar spinal stenosis symptoms.

	Acute pain (*n* = 29)							Chronic pain (*n* = 77)							
	Total (*n* = 29)				Type A (*n* = 3)/Type B (*n* = 8)/Type C (*n* = 18)			Total (*n* = 77)				Type A (*n* = 26)/Type B (*n* = 28)/Type C (*n* = 23)			
	Mean (SD)	Range	Median (IQR)	95% CI	Mean (SD)	Range	Median (IQR)	Mean (SD)	Range	Median (IQR)	95% CI	Mean (SD)	Range	Median (IQR)	*p* value
Age (years)	67.2 (14.1)	38–87	71 (60–79)	61.8–72.6	70.3 (10.6)/75.4 (8.4)/63.9 (15.5)	59–80/63–87/38–82	72 (65.5–76)/75.5 (70–81)/64.5 (49.5–79)	70.7 (13.6)	27–97	73 (66–80)	67.6–73.8	69.3 (14.8)/72.9 (12.7)/69.7 (13.6)	29–89/32–97/27–87	73 (58–81)/75 (69–83)/71 (66.5–78.5)	0.319^a^
Gender: no. (%)															
Male/female								37 (48.1)/40 (51.9)						<0.05 ^c^	
Height (m)	1.62 (0.11)	1.43–1.84	1.64 (1.56–1.68)	1.58–1.67	1.60 (0.12)/1.62 (0.08)/1.63 (0.13)	1.46–1.67/1.48–1.72/1.43–1.84	1.67 (1.57–1.67)/1.62 (1.57–1.68)/1.64 (1.55–1.71)	1.59 (0.10)	1.34–1.79	1.60 (1.51–1.67)	1.57–1.61	1.61 (0.10)/1.58 (0.10)/1.57 (0.11)	1.41–1.79/1.42–1.75/1.34–1.76	1.61 (1.53–1.69)/1.61 (1.48–1.65)	0.170^b^
Weight (kg)	65.7 (13.3)	42.8–105.8	63.0 (58.5–72.0)	60.6–70.8	57.2 (1.5)/62.0 (10.5)/68.7 (14.8)	56.2–59/46 - 78.8/42.8–105.8	56.5 (56.4–57.8)/62.7 (57.2–67)/68.1 (59.6–73.1)	63.8 (15.6)	37.0–128.4	60.8 (53.0–72.8)	60.3–67.3	69.7 (19.3)/61.5 (11.7)/69.0 (13.5)	47.5–128.4/37.0–82.5/37.9–93.8	65.9 (57.6–125)/59.2 (52.6–71.1)/58.0 (51.7–66.3)	0.545^a^
BMI	24.8 (3.8)	20.3–38.1	24.2 (22.2–26.7)	23.4–26.2	22.7 (3.3)/23.6 (2.2)/25.7 (4.2)	20.4–26.5/20.6–27.2/20.3–38.1	21.2 (20.8–26.5)/24 (22 - 24.5)/25 (23.7–27.4)	25.1 (5.0)	15.8–44.2	24.4 (22.3–26.8)	24.0–26.2	26.7 (6.0)/24.3 (4.2)/24.1 (4.2)	20.7–44.2/15.8–36.2/17.4–35.2	24.8 (22.8–28.3)/23.6 (22.0–26.7)/24.4 (22.3–25.7)	0.788^a^
Duration of LSS symptoms (months)	1.8 (0.9)	1–3	2 (1–3)	1.5–2.1	3.0 (0)/1.8 (0.9)/1.7 (0.8)	3 - 3/1–3/1–3	3 (3-3)/1.5 (1–2.3)/1 (1-2)	40.2 (51.2)	4–240	24 (12–36)	28.6–51.8	37.6 (56.6)/46.5 (61.2)/35.6 (27.2)	4–240/2–240/6–108	12 (12–36)/24 (10–39)/24 (12–43)	<0.001^a^
Classification of LSS by site of symptom appearance: no. (%)															<0.01^d^
Bilateral symptom	25 (86.2)				1 (33.3)/6 (75.0)/18 (100)			44 (57.1)				12 (46.2)/9 (32.1)/23 (100)			
Unilateral symptom	4 (13.8)				2 (66.7)/2(25.0)/0(0)			33 (42.9)				14 (53.8)/19 (67.9)/0 (0)			
Number of spinal stenosis	1.8 (0.7)	1–3	2 (1–2)	1.5–2.1	2.0 (1.0)/1.8 (0.7)/1.8 (0.7)	1–3/1–3/1–3	2 (1.5–2.5)/2 (1-2)/2 (1-2)	2.2 (0.9)	1–5	2 (2–3)	2.0 to 2.4	2.2 (1.0)/2.3 (0.9)/2.1 (0.9)	1–4/1–4/1–5	2 (1.3–3)/2 (2-3)/2 (2-2)	<0.05^a^
Location of spinal stenosis: no. (%)															0.130^d^
L1/2	0 (0)				0 (0)/0 (0)/0 (0)			4 (5.2)				0 (0)/1 (3.6)/3 (13.0)			
L2/3	3 (10.3)				1 (33.3)/1 (33.3)/1 (33.3)			19 (24.7)				1 (3.8)/8 (28.6)/10 (43.5)			
L3/4	14 (48.3)				2 (66.7)/4 (50.0)/8 (44.4)			45 (58.4)				12 (46.2)/19 (67.9)/14 (60.9)			
L4/5	20 (69.0)				3 (100)/5 (62.5)/12 (66.7)			66 (85.7)				23 (88.5)/25 (89.3)/18 (78.3)			
L5/S	17 (58.6)				2 (66.7)/4 (50.0)/11 (61.1)			26 (33.8)				2 (7.7)/12 (42.9)/12 (52.2)			
Grade of spinal stenosis according to Schizas: no. (%)															0.494 ^d^
A2	2(6.9)				0 (0)/0 (0)/2 (11.1)			3 (3.9)				0 (0)/2 (7.1)/1(4.3)			
A3	1(3.4)				0 (0)/0 (0)/1 (5.6)			1 (1.3)				1 (3.8)/0 (0)/0 (0)			
A4	1(3.4)				0 (0)/0 (0)/1 (5.6)			6 (7.8)				2 (7.7)/0 (0)/4 (17.4)			
B	3 (10.3)				0 (0)/1 (12.5)/2 (11.1)			5 (6.5)				3 (11.5)/1 (3.6)/1 (4.3)			
C	15 (51.7)				2 (66.7)/4 (50.0)/9 (50.0)			39 (50.6)				8 (30.8)/11 (39.3)/11 (47.8)			
D	7 (24.1)				1 (33.3)/3 (37.5)/3 (16.7)			32 (41.6)				12 (46.2)/14 (50.0)/6 (26.1)			
Conservative treatment: no. (%)															0.534 ^d^
None	1 (3.4)				0 (0)/0 (0)/1 (5.6)			4 (5.2)				1 (3.8)/1 (3.6)/2 (8.7)			
Medicine	26 (89.7)				3 (100)/7 (87.5)/16 (88.9)			55 (71.4)				19 (73.1)/22 (78.6)/14 (60.9)			
Infusion·injection	4 (13.8)				2 (66.7)/0 (0)/2 (11.1)			6 (7.8)				3 (11.5)/2 (7.1)/1 (4.3)			
Block	11 (37.9)				1 (33.3)/5 (62.5)/5 (27.8)			28 (36.4)				7 (26.9)/12 (42.9)/9 (39.1)			
Rehabilitation	4 (13.8)				2 (66.7)/1 (12.5)/1 (5.6)			25 (32.5)				8 (30.8)/8 (28.6)/9 (39.1)			
Massage	11 (37.9)				2 (66.7)/4 (50.0)/5 (27.8)			23 (29.9)				8 (30.8)/12 (42.9)/3 (13.0)			
Acupuncture	4 (13.8)				1 (33.3)/1 (12.5)/2 (11.1)			13 (16.9)				5 (19.2)/4 (14.3)/4 (17.4)			
NRS for LBP (0–10 range)	4.4 (3.0)	0–10	5 (2–7)	3.3 to 5.5	5.7 (3.2)/5.1 (3.6)/3.9 (2.7)	2–8/0–10/0–8	7 (4.5–7.5)/4.5 (2.7–6.0)/5.0 (1.3–6.0)	5.6 (2.7)	0–10	6 (4–8)	5.0–6.2	5.3 (3.3)/5.9 (2.4)/5.5 (2.3)	0–10/0–10/1–10	5.5 (3–8)/6.5 (4.8–8)/6 (4–7)	<0.05 ^a^
NRS for leg pain (0–10 range)	5.2 (2.8)	0–10	5 (3–8)	4.1–6.3	7.7 (0.6)/4.5 (3.2)/5.1 (2.6)	7-8/0–10/0–9	8 (7.5–8.0)/4 (2.8–5.6)/5 (3.5–6.8)	5.0 (3.0)	0–10	5 (3–8)	4.3–5.7	5.1 (3.5)/5.0 (2.9)/5.1 (2.5)	0–10/0–10/1–10	5 (2.3–8.0)/5 (3.0–7.3)/5 (3–7)	0.881 ^a^
NRS for leg numbness (0–10 range)	4.6 (3.1)	0–10	4 (2–7)	3.4–5.8	7.3 (0.6)/5.1 (3.6)/3.8 (3.0)	7 - 8/0–10/0–9	7 (7.0–7.5)/4 (2.8–8.3)/3 (1.3–6.8)	4.6 (3.3)	0–10	5 (1–8)	3.9–5.3	4.2 (3.6)/5.3 (2.8)/4.1 (3.3)	0–10/0–10/0–10	3 (1–8)/5 (4–6.3)/4 (1–7)	0.978 ^a^
painDETECT (−1–38 range)	13.9 (6.3)	3–25	13 (8–18)	11.5–16.3	18.3 (10.7)/12.6 (6.5)/13.2 (5.5)	6–25/5–24/3–23	24 (15.0–24.5)/11 (7.8–17.3)/13.5 (8.8–16.8)	13.5 (6.1)	0–33	12 (10–18)	12.1–14.9	12.7 (6.6)/14.6 (6.1)/13.3 (5.7)	4–33/0–25/2–27	11.5 (9.0–14.8)/16 (10–19)/12 (9.5–17.0)	0.949 ^b^
Nociceptive pain: no. (%)	14 (48.3)				1 (33.3)/5 (62.5)/8 (44.4)			39 (50.6)				15 (57.7)/12 (42.9)/12 (52.2)			
Type of unclear: no. (%)	9 (31.0)				0 (0)/2 (25)/7 (38.9)			23 (29.9)				8 (30.8)/8 (28.6)/7 (30.4)			
Neuropathic pain: no. (%)	6 (20.7)				2 (66.7)/1 (12.5)/3 (16.7)			15 (19.5)				3 (11.5)/8 (28.6)/4 (17.4)			
CSI (0–100 range)	23.0 (13.3)	2–51	23 (14–30)	17.9–28.1	33.7 (12.9)/20.4 (12.4)/22.4 (13.6)	23–48/2–38/3–51	30 (26.5–39.0)/20.5 (11.3–30.3)/21 (14.5–30.0)	24.9 (13.4)	2–63	23 (16–33)	21.9 to 27.9	21.3 (14.0)/27.6 (13.4)/25.4 (12.3)	2–63/3–52/7–54	18.5 (10.5–28.8)/31 (17.8–37.3)/25 (18–32)	0.533 ^a^
High CSI: no. (%)	3 (10.3)				1 (33.3)/0 (0)/2 (11.1)			8 (10.4)				2 (7.7)/4 (14.3)/2(8.7)			
Low CSI: no. (%)	26 (89.7)				2 (66.7)/8 (100)/16(88.9)			69 (89.6)				24 (92.3)/24 (85.7)/21 (91.3)			
ZCQ (12−55 range)	34.7 (5.5)	22–45	36 (30–39)	32.6–36.8	40.7 (3.8)/35.4 (5.7)/33.4 (5.2)	38–42/28–39.3/22–36.8	39 (38.5–42.0)/37 (29.8–39.3)/34 (29.3–36.8)	33.2 (7.0)	13–51	34 (29–38)	31.6 to 34.8	32.4 (7.9)/35.1 (6.2)/31.9 (6.5)	13–51/18–48/19–44	32.5 (27.5–36.8)/36 (30–39)/32 (27.0–36.5)	0.313 ^b^
Symptom severity (7–35)	22.7 (3.8)	15–31	22 (20–25)	21.3–24.1	24.0 (3.6)/23.9 (4.5)/21.9 (3.5)	20–27/19–31/15–29	25 (22.5–26.0)/22.5 (20.8–26.3)/20 (20.0–24.8)	21.0 (4.6)	8–32	21 (18–24)	20.0–22.0	20.7 (5.4)/22.0 (4.1)/20.2 (4.2)	8–32/11–29/10–28	20.5 (17–24)/22.5 (19–25)/20 (17.5–23)	0.112 ^b^
Physical function (5–20 range)	12.0 (3.5)	6–19	12 (10–15)	10.7 to 13.3	16.7 (3.2)/11.5 (3.4)/11.5 (3.1)	13–19/6–17/6–16	18 (15.5–18.5)/11.5 (9.8–13.3)/11.5 (9.3–14.8)	12.2 (3.1)	5–19	12 (10–14)	11.5–12.9	11.7 (3.1)/13.1 (3.0)/11.7 (3.2)	5–19/7–19/5–17	12 (10.0–13.8)/13 (10.8–15.0)/12 (9.5–14.0)	0.754 ^b^
EQ-5D-5L (−0.025–1 range)	0.496 (0.247)	0.041–0.873	0.534 (0.288–0.695)	0.402–0.590	0.282 (0.190)/0.588 (0.299)/0.491 (0.250)	0.089–0.469/0.109–0.873/0.041–0.808	0.288 (0.188–0.379)/0.615 (0.513–0.707)/0.549 (0.244–0.691)	0.520 (0.209)	0.035–0.873	0.355 (0.035–0.648)	0.473–0.567	0.519 (0.205)/0.502 (0.221)/0.544 (0.205)	0.060–0.808/0.035–0.873/0.041–0.873	0.581 (0.412–0.683)/0.549 (0.327–0.634)/0.583 (0.484–0.648)	0.809 ^b^

SD: standard deviation; CI: confidence interval; NRS: Numerical Rating Scale; CSI: Central Sensitization Inventory; ZCQ: Zurich Claudication Questionnaire; EQ-5D-5L: EuroQol-5 dimensions-5 levels. The range represents the minimum and maximum values. IQR represents the interquartile range. Acute pain and chronic pain were compared. ^a^According to the *t*-test; ^b^according to the Mann–Whitney *U* test; ^c^according to the chi-square test; ^d^according to Fisher's exact probability test.

**Table 3:** A matrix of the correlations.

	1	2	3	4	5	6	7	8	9	10	11	12	13	14	15	16	17	18	19	20	21
(1) Age	—																				
(2) Gender	0.174	—																			
(3) Height	−0.403^*∗∗*^	−0.717^*∗∗∗*^	—																		
(4) Weight	−0.393^*∗∗∗*^	−0.479^*∗∗∗*^	0.664^*∗∗∗*^	—																	
(5) BMI	−0.193^*∗*^	−0.028	0.031	0.732^*∗∗∗*^	—																
(6) Duration of LSS symptoms	0.130	0.309^*∗∗∗*^	−0.227^*∗∗∗*^	−0.159	−0.020	—															
(7) Classification of pain by course	0.089	0.218^*∗*^	−0.134	−0.090	−0.007	0.777^*∗∗∗*^	—														
(8) Classification of LSS by site of symptom appearance	0.264^*∗∗∗*^	−0.069	0.000	−0.034	−0.079	0.154	0.272^*∗∗*^	—													
(9) Clinical phenotype of LSS	−0.090	0.060	−0.040	−0.047	−0.034	−0.181	−0.310^*∗∗∗*^	−0.511^*∗∗∗*^	—												
(10) Number of spinal stenosis	0.135	0.038	−0.118	−0.195^*∗*^	−0.162	0.166	0.177	0.253^*∗∗*^	−0.105	—											
(11) Grade of spinal stenosis according to Schizas	−0.009	−0.064	0.015	−0.087	−0.122	0.009	0.140	0.248^*∗∗*^	−0.212∗	0.366^*∗∗∗*^	—										
(12) Number of conservative treatment	−0.105	0.068	−0.051	−0.001	0.021	0.137	0.178	0.065	−0.104	0.157	0.147	—									
(13) NRS for LBP	0.114	−0.011	0.029	0.003	0.001	0.218^*∗*^	0.191^*∗*^	0.044	−0.124	0.021	0.008	0.260^*∗∗*^	—								
(14) NRS for leg pain	0.068	−0.108	0.084	−0.056	−0.17	−0.021	−0.013	0.052	−0.039	0.145	0.053	0.242^*∗∗∗*^	0.464^*∗∗∗*^	—							
(15) NRS for leg numbness	0.045	−0.187^*∗*^	0.193	0.148	0.029	0.014	0.0007	0.091	−0.081	0.041	0.071	0.183	0.387^*∗∗∗*^	0.662^*∗∗∗*^	—						
(16) painDETECT	−0.078	−0.075	0.117	0.037	−0.035	0.133	0.007	0.068	0.032	0.126	0.006	0.229^*∗∗*^	0.341^*∗∗∗*^	0.428^*∗∗∗*^	0.429^*∗∗∗*^	—					
(17) CSI	0.086	0.042	−0.071	−0.081	−0.04	0.186∗	0.057	0.172	0.042	0.030	−0.167	0.281^*∗∗*^	0.444^*∗∗∗*^	0.327^*∗∗∗*^	0.293^*∗∗∗*^	0.550^*∗∗∗*^	—				
(18) ZCQ	0.099	−0.141	0.079	−0.048	−0.161	−0.030	−0.099	0.272^*∗∗*^	−0.085	0.061	0.128	0.171	0.379^*∗∗∗*^	0.534^*∗∗∗*^	0.463^*∗∗∗*^	0.469^*∗∗∗*^	0.349^*∗∗∗*^	—			
(19) ZCQ for symptom severity	0.086	−0.194^*∗*^	0.170	−0.000	−0.158	−0.070	−0.155	0.239^*∗∗*^	−0.040	0.062	0.180	0.171	0.336^*∗∗∗*^	0.504^*∗∗∗*^	0.492^*∗∗∗*^	0.523∗∗∗	0.307^*∗∗∗*^	0.877^*∗∗∗*^	—		
(20) ZCQ for physical function	0.130	0.007	−0.100	−0.141	−0.162	0.051	0.031	0.224^*∗*^	−0.059	0.107	0.050	0.149	0.319^*∗∗∗*^	0.420^*∗∗∗*^	0.268∗∗	0.308^*∗∗∗*^	0.305^*∗∗∗*^	0.802^*∗∗∗*^	0.453^*∗∗∗*^	—	
(21) EQ-5D-5L	−0.126	0.089	0.028	0.091	0.122	−0.061	0.024	−0.100	0.065	−0.029	−0.084	−0.270^*∗∗*^	−0.451^*∗∗∗*^	−0.519^*∗∗∗*^	−0.344^*∗∗∗*^	−0.426^*∗∗∗*^	−0.461^*∗∗∗*^	−0.641^*∗∗∗*^	−0.472^*∗∗∗*^	−0.640^*∗∗∗*^	—

BMI: body mass index; NRS: Numerical Rating Scale; CSI: Central Sensitization Inventory; ZCQ: Zurich Claudication Questionnaire; EQ-5D-5L: EuroQol-5 dimensions-5 levels. ^*∗*^*p* < 0.05; ^*∗∗*^*p* < 0.01; ^*∗∗∗*^*p* < 0.001.

**Table 4:** Multiple regression analysis using painDETECT as dependent variable.

	Partial regression coefficient	Standard error	95% CI	*β*	*t*	*p* value
Age	−0.026	0.034	−0.093–0.041	−0.058	−0.769	0.444
Gender	−0.573	0.938	−2.435–1.288	−0.046	−0.611	0.543
Classification of LSS by site of symptom appearance	−0.625	0.990	−2.591–1.340	−0.048	−0.631	0.529
NRS for leg pain	0.276	0.188	−0.097–0.649	0.130	1.466	0.146
CSI	0.216	0.037	0.143–0.289	0.468	5.893	<0.001
ZCQ for symptom severity	0.419	0.127	0.167–0.671	0.304	3.304	<0.001
ZCQ for physical function	0.012	0.167	−0.319–0.343	0.006	0.070	0.945

CI: confidence interval; NRS: Numerical Rating Scale; CSI: Central Sensitization Inventory; ZCQ: Zurich Claudication Questionnaire. *R*^2^ adj. = 0.478.

## Data Availability

The datasets generated and analyzed during the current study are available from the corresponding author on reasonable request.
